# A novel multicolor flow-cytometry application for quantitative detection of receptors on vascular smooth muscle cells

**DOI:** 10.1371/journal.pone.0186504

**Published:** 2017-10-30

**Authors:** Aneta Radziwon-Balicka, Matilda Degn, Sara E. Johansson, Karin Warfvinge, Lars Edvinsson

**Affiliations:** 1 Department of Clinical Experimental Research, Glostrup Research Institute, Rigshospitalet, Glostrup, Denmark; 2 Department of Clinical Sciences, Division of Experimental Vascular Research, Lund University, Lund, Sweden; Medical University Innsbruck, AUSTRIA

## Abstract

There is a need to develop new techniques for quantitative measurement of receptors expression on particular vasculature cells types. Here, we describe and demonstrate a novel method to measure quantitatively and simultaneously the expression of endothelin _B_ receptor (ET_B_) on vascular smooth muscle cells (VSMC). We isolated cells from male rat tissues such as: brain pial, brain intraparenchymal and retina vessels. To analyze solid tissues, a single-cell suspension was prepared by a combined mechanic and enzymatic process. The cells were stained with Fixable Viability Dye, followed by fixation, permeabilization and antibodies staining. The expression of ET_B_ receptors on VSMC was measured by flow-cytometry and visualized by fluorescence microscopy. We obtained a high percentage of viable cells 87.6% ± 1.5% pial; 84.6% ± 4.3% parenchymal and 90.6% ± 4% retina after isolation of single cells. We performed a quantitative measurement of ET_B_ receptor expression on VSMC and we identified two subpopulations of VSMC based on their expression of smooth muscle cells marker SM22α. The results obtained from pial vessels are statistically significant (38.4% ± 4% vs 9.8% ± 3.32%) between the two subpopulations of VSMC. The results obtained from intraparenchymal and retina vessels were not statistically significant. By specific gating on two subpopulations, we were able to quantify the expression of ET_B_ receptors. The two subpopulation expressed the same level of ET_B_ receptor (p = 0.45; p = 0.3; p = 0.42) in pial, parenchymal and retina vessels, respectively. We applied our method to the animals after induction of subarachnoid hemorrhage (SAH). There was statistically significant expression of ET_B_ receptor (p = 0.02) on VSMC between sham 61.4% ± 4% and SAH 77.4% ± 4% rats pial vessels. The presented technique is able to quantitatively and selectively measure the level of protein expression on VSMC. The entire technique is optimized for rat tissue; however the protocol can also be adapted for other species.

## Introduction

Cerebral blood flow and metabolism are constantly at high levels and the richly vascularized brain receives about 20% of cardiac output at rest. The arteries, arterioles and veins contain three main layers such as *tunica intima*, *tunica media* and *tunica adventitia*, however to different degrees. The tunica intima is luminally covered by a single layer of endothelial cells [1]. The tunica media contains mainly smooth muscle cells, which regulate the vascular tone in the blood vessels [2]. The tunica adventitia is made by connective tissue, nerves and some fibroblasts. However, tissue for quantitative protein analysis with the gold standard western blot method will inevitably contain all three layers. However there is uncertainty on the localization and degree of receptor expression. We have focused on expressional changes in the vasculature in stroke as one mechanism that is accessible and has a key role [3]. This work utilizes many molecular biological methods, however, the amount of tissue available for these experiments is sparse hence the development of sensitive methods for protein analysis is needed. In order to determine pathology of the vascular diseases, it is necessary to develop a method, which will quantitatively measure expressions of the receptors/proteins on cells of interest. Methods describe very early isolation of cells by mechanical dissociation and using cells filters with different pore sizes [4]. Later protocols introduced low- and high- speed density gradient centrifugation. The changes in protein expression and DNA damage is resulted by shear stress during high-speed centrifugation [5]. Recently, the protocols presented enzymatic cocktails, which effect will depend on the ingredients [6]. Here, we proposed a combined technique of mechanical, enzymatic and density gradient centrifugation to achieve high and reproducible yield levels of viable cells after isolation from CNS vasculature. This study focuses on how to isolate viable single cell populations of smooth muscle cells from different tissue origin and measure quantitatively the endothelin B protein (ET_B_) expression by flow-cytometry. We used the robust changes seen after experimental subarachnoid hemorrhage (SAH) stroke of the endothelin B receptor (ET_B_) for validation of our new method.

## Materials and methods

### Reagents

Anti-SM22 (1:100, ab10135, Abcam, UK), Goat isotope control IgG (5 μg/mL, ab18433 Abcam), Rabbit anti-ET_B_ receptor (1:100, ab117529, Abcam), Rabbit isotope control IgG (10 μg/mL, ab18433, Abcam), Allophycocyanin (APC)-conjugated donkey anti-rabbit IgG (1:100, Jackson ImmunoResearch), Alexa 488-conjugated donkey anti-goat IgG (1:100, Jackson ImmunoResearch), Fixable Viability Dye eFluor 780 (eBioscience), Liberase TM Research Grade (Roche), German coverslip with Poly-L-lysine coating on both sides cat no H-12-pII Neuvitro, USA, Vectashield Antifade Mounting Medium (Vector Laboratories, USA), BSA (Bovine Serum Albumin, Sigma Aldrich), FBS (Fetal Bovine Serum, Sigma Aldrich), Triton X-100 (Sigma Aldrich).

### Animals

We used male Wistar rats (10–12 weeks, 300–370 g, Taconic, Denmark). For the SAH male Sprague-Dawley rats (300–350 g, Taconic, Denmark) were used. The animals were located in an accredited Animal facility at Glostrup Research Institute, Rigshospitalet, Glostrup, Denmark. The animals were provided with standard chow and water *ad libitum*. The animals were kept at 12 hours light and 12 hours dark cycles. All animal experiments and surgical procedure were performed in accordance with the EU directive 2010/63/EU, Danish Animal Inspectorate (2012-15-2934-389) and ARRIVE guidelines. The study was approved by the Danish Animal Inspectorate. The animals were stunned with CO_2_ (70%)/O_2_ (30%) and euthanized by decapitation. The brain and eyes were subsequently removed and placed in a cold +4° C phosphate-buffered saline (PBS) buffer solution. The basilar (BA) and middle cerebral arteries (MCA) from brain vasculature were carefully dissected out under an operating microscope. The retinas were carefully removed from the eyeballs by using surgical tweezers.

### Preparation of single-cell suspensions

The MCA and BA were isolated and pooled from one animal (pial vasculature). The intraparenchymal vasculature was isolated from the forebrain by first removing the pial vasculature. The forebrain structures were obtained by first discarding the hind part of the brain (cerebrum behind MCA and cerebellum). The retinal tissue from the eyes of each animal were isolated and pooled. The retina vasculature was obtained by carefully removing the retina layer from the rest of eye tissue. The isolation of single cells population was performed for flow-cytometry analysis: pial vasculature; parenchymal vasculature and retina vasculature (described below separately).

#### Pial vasculature

Vascular smooth muscle cells (VSMCs) from the MCA and BA were isolated by placing the tissue to enzymatic digestion solution at the concentration of 1.25 mg/ml, which contains highly purified Collagenase I and Collagenase II in a medium of Thermolysin (Liberase TM Research Grade, Roche) for 1.5 hour at 37°C in the thermomixer (Eppendorf) at 500 g. To improve digestion of the tissue, the suspension was pipetted up and down every 30 min during the process. The best time to stop the enzymatic digestion is when the cellular suspension is homogeneous without visible fragments. The digestion process was stopped by physiological buffered saline PBS+5% bovine serum albumin (BSA, Sigma Aldrich). The samples were sieved through 100 μm cell strainer to separate single cells and remove undigested cell clumps. The cells suspension was rinsed with ice-cold PBS+5% BSA. Collected cells were centrifuged at 900 g for 10 min at room temperature and the cells were washed twice in PBS. 1μL of Fixable Viability Dye eFluor 780 (FVD, eBioscience) was added to the cells of concentration 1-10x10^6^/ml, immediately vortexed and incubated for 30 min at +2–8°C, protected from light. The FVD is used to irreversibly label dead cells prior to fixation and permeabilization procedures. The isolated cells suspension was fixed with 4% paraformaldehyde (PFA) for 30 min and then the cells were washed twice by PBS for further analysis.

#### Parenchymal vasculature

In order to receive VSMCs from the parenchymal vasculature first the tissue of forebrain without pial vasculature was disrupted mechanically by surgical scalpel. Next, the tissue was placed to enzymatic digestion solution such as Liberase TM Research Grade (Roche) at the concentration of 1.25 mg/ml for 1.5 hour at 37°C in the thermomixer at 500 g. The suspension was pipetted up and down every 30 min. during the process. The digestion process was stopped by PBS+5% BSA. The samples were sieved through 100 μm cell strainer to remove undigested cell clumps and separate single cells. The cells suspension was rinsed with ice-cold PBS+5% BSA. Collected cells were centrifuged at 900 g for 10 min at room temperature. The various cells from brain tissue were resuspended in 10 ml of PBS+5% FBS (Fetal bovine serum, Sigma Aldrich) and carefully layered on 7.5 ml of Ficoll-Paque PLUS (GE Healthcare, Denmark) in 50 ml centrifuge tube. The different cells were separated by a centrifugation for 30 min at 400 g, room temperature, without breaks. During these step viable cells such as neurons, glial cells and endothelial, smooth muscle cells were separated from red blood cells and cellular debris.

After centrifugation, the samples were kept on ice. The interphase containing viable cells were transferred into a clean tube and 2 volumes of PBS+5% FBS was added. In order to remove Ficoll-Paque PLUS the samples were centrifuged at 600 g for 7 min at room temperature. Next, the cells were washed twice in PBS. The cells were stained with FVD and fixated with 4% PFA as previously described.

#### Retina vasculature

First, the retina tissue was disrupted mechanically by surgical scalpel. Next, the tissue was subjected to enzymatic digestion solution with Liberase TM Research Grade at the concentration of 1.25 mg/ml for 1.5 hour at 37°C in the thermomixer at 500 g. The suspension was pipetted up and down every 30 min during the process. The digestion process was stopped by adding PBS+5% BSA. The samples were sieved through 70 μm cell strainer to remove undigested cell clumps and separate single cells, and then rinsed with cold PBS+5% BSA. The cells were collected, stained with FVD and fixated with PFA, as previously described.

#### Intracellular flow-cytometry

Intracellular flow-cytometry was performed in order to investigate the expression of ET_B_ receptor on vascular smooth muscle cells within pial vessels (BA and MCA), parenchymal vessels and retina. 1 mL of 0.25% Triton X-100 (Sigma Aldrich) in PBS buffer was added to cells pellets in order to permeablize cells membrane. The samples were incubated for 30 minutes at room temperature with rocking, protected from light and washed once with 1 mL of PBS. Next, the cells were resuspended in blocking buffer containing 5% normal donkey serum in PBS and incubated for 2 hours at room temperature with rocking, protected from light. The cell suspensions were stained overnight at +4°C temperature with primary goat anti-SM22α (1:100, Abcam, UK) or goat isotope control IgG (5 μg/mL, Abcam, UK) in blocking buffer (PBS+5% normal donkey serum) protected from light with rocking, respectively. Excess of primary antibody was removed by washing twice with 1 mL of PBS. Subsequently, the cell samples were incubated with Alexa 488-conjugated donkey anti-goat IgG (1:100, Jackson ImmunoResearch) for 2 hours at room temperature, in the dark with rocking. Excess of secondary antibody was removed by washing twice with PBS. Next, cells samples were incubated with primary antibodies rabbit anti-ET_B_ (1:100, Abcam, UK) or rabbit isotope control IgG (10 μg/mL, Abcam, UK) in blocking buffer overnight at +4°C temperature with rocking, protected from light, respectively. The cell samples were washed twice with 1 mL of PBS and incubated with Allophycocyanin (APC)-conjugated donkey anti-rabbit IgG (1:100, Jackson ImmunoResearch) for 2 hours at room temperature, in the dark with rocking. Finally, the cells were once again washed twice with PBS and then diluted to a final volume of 0.5 mL with PBS before analysis by flow-cytometry. To identify ET_B_ receptors on VSMC flow-cytometry was performed. The stained cells suspension was analyzed by fluorescent-activated cell sorting (FACS) on the BD FACSVerse machine (BD Biosciences, USA). Fluorescence was excited with a 488 nm blue laser and 640 nm red laser. First the viable cells negative for FVD 780 were gated and future analyzed. The population of SM22α-positive cells was identified and from which a sub-gate was set in order to detect ET_B_ receptor expression on SM22α-positive cells. The ratio of SM22α-positive cells expressing ET_B_ was calculated for each sample. Data was analyzed with the BD FACSuite Software.

### Immunofluorescence microscopy

For immunofluorescence microscopy, the vascular smooth muscle cells were isolated from pial, parenchymal and retina vasculature as described above. The cell suspension were fixed for 30 min in 4% formaldehyde in PBS and washed twice with PBS buffer. The fixed cells were cytospinned onto poly-L-lysine coated coverslips (Neuvitro, USA) at 250 g for 5 min. The cells were permeabilized with PBS buffer containing 0.25% Triton X-100. Next, the samples were blocked for 2 hours in PBS with 5% normal donkey serum at room temperature with rocking. Followed by the incubation with primary goat anti-SM22α (1:100, Abcam, UK) or goat isotope control IgG (5 μg/mL, Abcam, UK) overnight at +4°C with rocking, respectively.

Coverslips were washed twice with PBS, treated with appropriate secondary antibody (Alexa 488-conjugated donkey anti-goat IgG, 1:100, Jackson ImmunoResearch) for 2 hours at room temperature, in the dark with rocking and washed twice with PBS. Subsequently, coverslips were incubated with rabbit anti-ET_B_ (1:100, Abcam, UK) or rabbit isotope control IgG (10 μg/mL, Abcam, UK) antibodies in the manner described earlier. Controls for the secondary antibodies were treated in the same fashion except for omission of the primary antibody.

The samples were mounted in Vectashield antifade mounting medium (Vector Laboratories, USA) and analyzed at room temperature on a fluorescence microscope (Nikon D-Eclipse C1, Tokyo, Japan) combined with a Nikon C1-SHV camera. Electronic shutters and image acquisition were under the control of NIS-Elements BR 3.1 software. Images were acquired by fluorescence microscopy with an image capture time of 50 to 100 milliseconds. Fluorescence was excited with the 480 nm and 543 nm laser lines. Images were merged together to determine co-localization. The images were then processed using Leica LAS AF Lite software. Finally, images were equally adjusted for contrast and brightness.

### In vivo subarachnoid hemorrhage

The SAH was induced as described before [7]. Male Sprague-Dawley rats were anesthetized by 2.5 ml/kg mixture of hypnorm-midazolam (1:1:2) in sterile water. Blood samples were regularly analysed and body temperature was kept at 37°C. Mean arterial blood pressure (MABP) and intracranial pressure (ICP) were continuously measured via catheters inserted into the tail artery and the cisterna magna, respectively. A laser- Doppler blood flow meter probe was placed on the dura through a hole in the skull drilled 4 mm anterior from bregma and 3 mm rightwards of the midline. Through a second hole drilled 6.5 mm anterior to bregma in the midline, a 27G blunt cannula was descended stereotactically at an angle of 30° to the vertical plane towards a final position of the tip immediate anteriorly to the chiasma opticum. After 30 minutes of equilibration, 300 μl of blood was withdrawn from the tail catheter and injected to the chiasma opticum. The pressure and rate of the blood injections was carefully controlled aiming at raising ICP to the higher range of mean MABP levels in all animals (app. 120 mmHg).

At the end of the procedure, the ICP catheter was cut and sealed 0.5 cm from the tip. The tail catheter, needle and laser- Doppler probe were removed and incisions closed. Rats were revitalized and extubated. After surgery the rats were recovered and received at the end of surgery and every 24 hours thereafter rats received subcutaneous injections of Carprofen (4 mg/kg) and 15 ml isotonic saline. Carprofen is a non-steroidal anti-inflammatory analgesic drug used here due to its long-lasting analgesic effect. Sham operated rats went through the same procedure with the exception that no blood was injected intracisternally. After 48 hours, the animals were stunned with 70% CO_2_/30% O_2_ and euthanized by decapitation.

### Statistics

Statistics were performed using Graph Pad Prism 5.0. All means are reported with SEM. The unpaired Student's t-tests (two-sided) were performed. A P-value less than 0.05 was considered significant. Each N was obtained from one animal (pial tissue was contained of one BA and two MCA; parenchymal tissue was contained of one forebrain without pial vasculature and retina tissue was contained of two retinas from the same animal). The single cells suspension was directly stained (not cultured) with antibodies and analyzed by flow-cytometry after isolation procedure of tissue.

## Results

**Fig 1:**
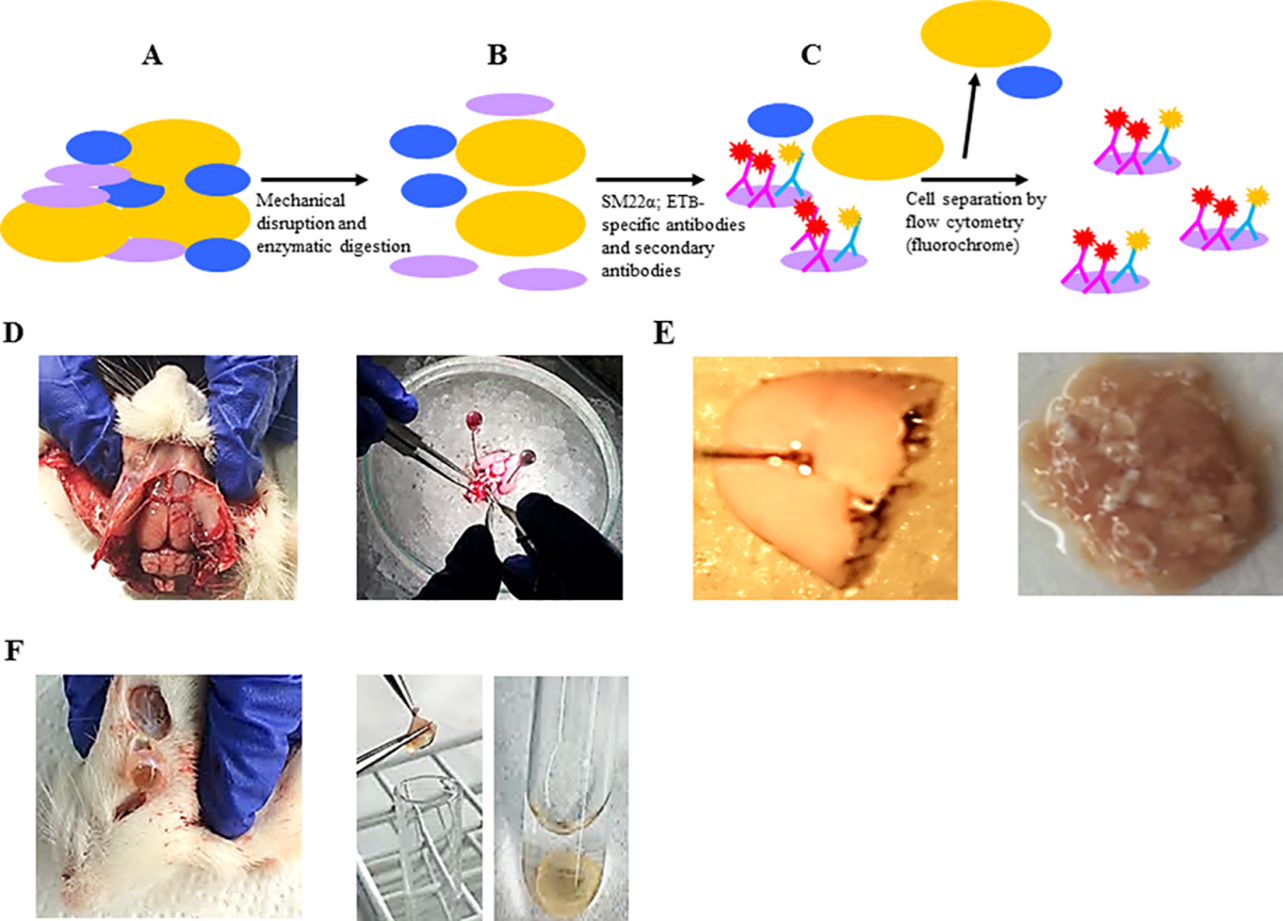
Schematic representation of the isolation and detection of smooth muscle cells from tissue by flow- cytometry. (A) The tissue is mechanically disrupted followed by enzymatic digestion to obtain single cells suspensions. (B) The cells are fixed, permeabilized and labeled by primary antibodies SM22α (VSMCs marker) and ET_B_ receptor followed by secondary antibodies. (C) The flow-cytometry protocol was developed to detect VSMC and their expressions of ET_B_ receptor. (D) Brain and pial vessels (middle cerebral arteries and basilar artery) isolation from rat. (E) The parenchymal vessels were isolated from the forebrain and the pial vessels were removed. (F) The retina layer isolation from the rest of rat eyes tissue.

We established a method for quantitative detection of ET_B_ receptor on vascular smooth muscle cells from solid rat tissue. A single-cell suspension was prepared by a mechanical and enzymatic process (Fig 1A). The cells were fixed by paraformaldehyde and stained sequentially with SM22α, ET_B_-specific antibodies and secondary antibodies (Fig 1B). Flow cytometry was performed in order to separate cells of interest (Fig 1C). We developed protocols to isolate single cells from pial (intracranial), parenchymal and retina vessels. First, we isolated the brain from the male Wistar rat then we isolated MCA and BA (Fig 1D). Next, the pial vasculature from the forebrain structures of brain was discarded and the tissue was minced by scalpel (Fig 1E). The retina vasculature was obtained by isolation of the rat’s eye. The cornea, lens and vitreous body were removed and retina cap was pulled out from the rest of the eye connective tissue (Fig 1F).

### Flow-cytometry

**Fig 2:**
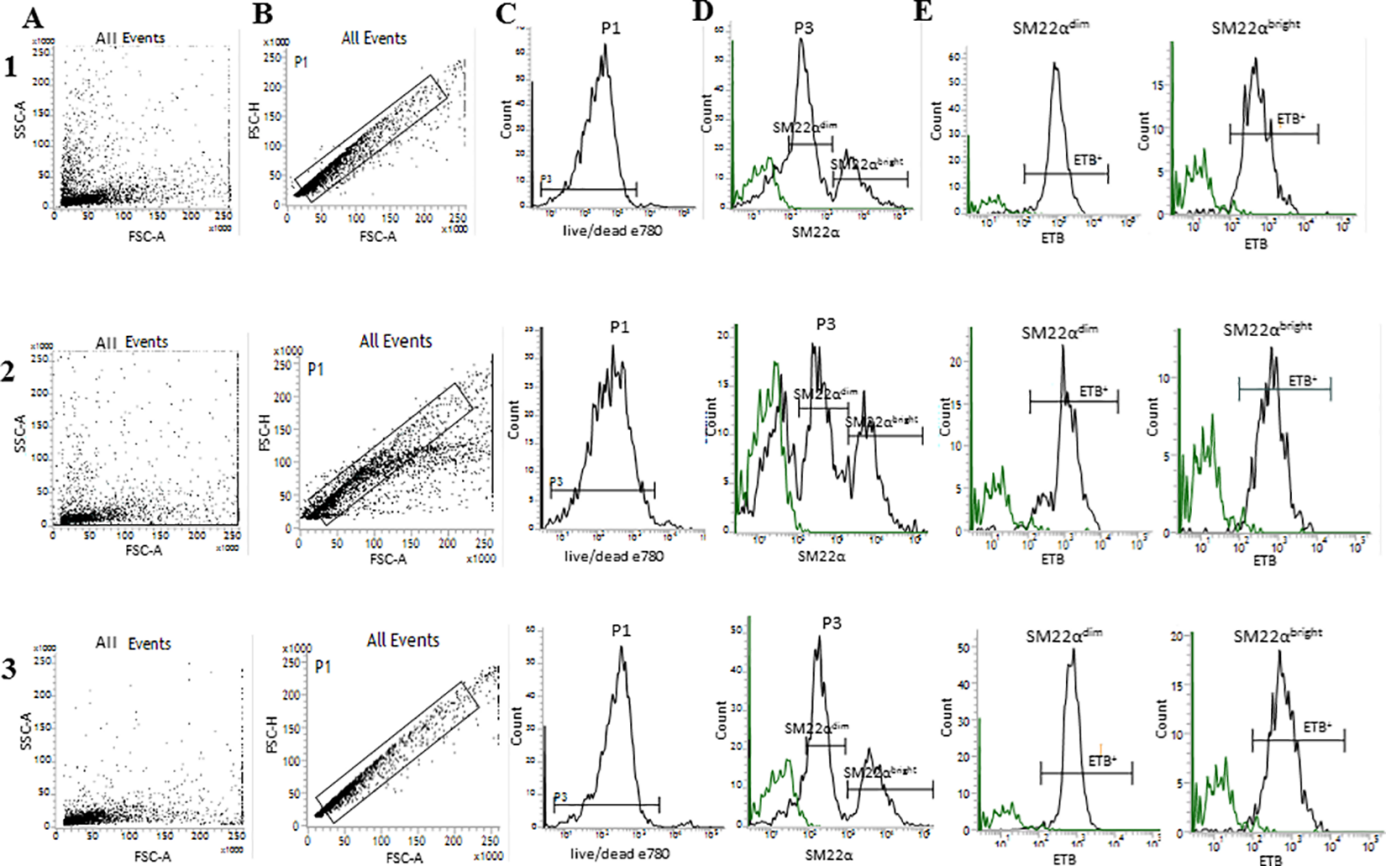
Representative dot plots histograms of the experimental setup and analysis of flow cytometry data. (A) Dot plot histogram for entire cell population after isolation. (B) Dot plot histogram of single cells population according to FSC-H (H- high) vs. FSC-A (A- area scaling). (C) Representative histogram of negative single cells population for Fixable Viability Dye eFluor 780 (viable cells, live/dead); (N = 6 pial, N = 4 parenchymal and N = 4 retina). (D) Representative histogram demonstrating two subpopulations of SM22α-positive events of viable cells suspension (log scale) (N = 5, *p˂0.05 pial, N = 4 parenchymal and N = 3 retina). (E) Representative histograms demonstrating ET_B_-positive events (log scale); (N = 5 pial, N = 4 parenchymal and N = 3 retina). The green line indicates the IgG for SM22α or ET_B_ respectively. The analysis was performed from three tissue types: 1- pial vessels, 2- cerebral parenchymal vessels and 3- retina vessels.

**Fig 3:**
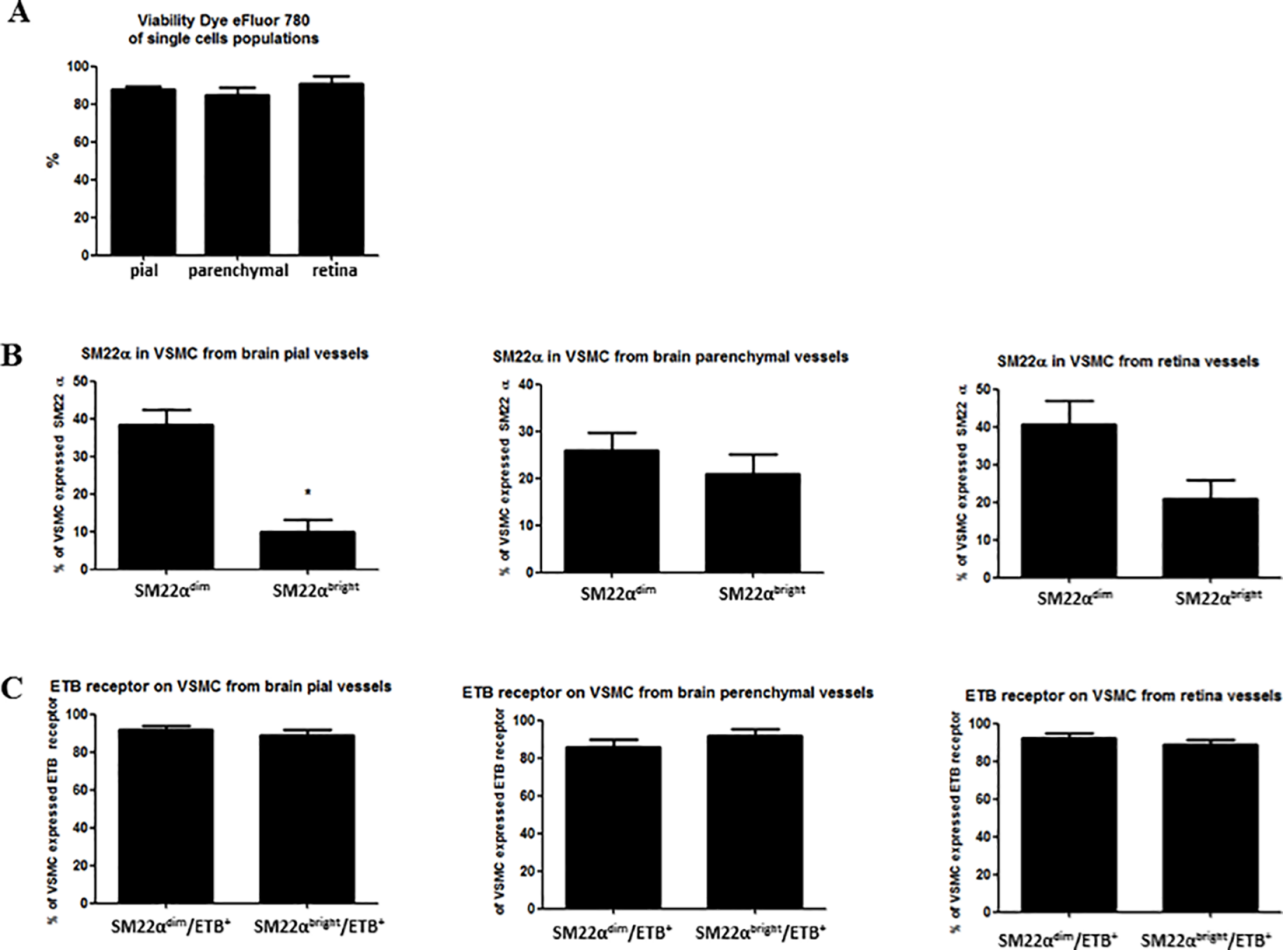
The bar graphs show summary data of flow-cytometry analysis. (A) The percentage of viable single cells after tissue isolation of pial, parenchymal and retina vessels (N = 6, N = 4 and N = 4, respectively). (B) The percentage of VSMC expressed SM22α from pial (N = 5, *p˂0.05), parenchymal (N = 4) and retina vessels (N = 3). (C) The percentage of VSMC (SM22α-positive) expressed ET_B_ receptor from pial, parenchymal and retina vessels (N = 5, N = 4 and N = 3 respectively).

We established a SM22α-specific flow-cytometry protocol to measure expression of ET_B_ receptors on VSMC on the cerebral and retina vasculature. Using this protocol, we identified VSMCs of two subpopulations based on their expression of marker protein (SM22α). By specific gating on a two subpopulations of VSMC, we were able to quantify the expression of ET_B_ receptors separately. We found that two subpopulations of VSMC, SM22α^dim^ and SM22α^bright^ expressing the same percentage of ET_B_ receptor. The cell suspensions of fresh rat brain (1) pial (intracranial) vessels, (2) parenchymal arterioles and (3) retina arterioles tissue are triplet stained with FVD, primary antibodies SM22α and ET_B_ receptors. Within the flow-cytometer the entire cell population of a given sample was detected according to cell size and granularity as seen in the representative forward scatter vs side scatter plot in Fig 2A. In order to discriminate doublets, the FSC-H (H-high) vs. FSC-A (A-Area Scaling) was correlated to detect disproportions between cell sizes vs. cell signal (Fig 2B). First, the single cells population was detected by negative signal of FVD, which indicated viable cells (Fig 2C). FVD negative events (viable single cells) were further sub-gated and SM22α and ET_B_ receptor were detected based on their fluorescence (Fig 2D). Furthermore, SM22α-positive events were further sub-gated and ET_B_ receptor expression was measured (Fig 2E). The bar graph on Fig 3A shows summary data of method reproducibility in the scope of viability of single cells (pial, parenchymal and retina vessels). We received 87.6% ± 1.5% (N = 6) pial; 84.6% ± 4.3% (N = 4) parenchymal and 90.6% ± 4% (N = 4) retina of viable cells after isolation process from different solid tissue types. Fig 3B shows VSMC of two subpopulations (SM22α^dim^ and SM22α^bright^) based on their expression of marker protein (SM22α). The results obtained from pial (intracranial) vessels are statistically significant (SM22α^dim^ 38.4% ± 4% vs SM22α^bright^ 9.8% ± 3.3%, N = 5, p = 0.0006). The results obtained from parenchymal vessels and retina vessels are not statistically significant (parenchymal vessels: SM22α^dim^ 26% ± 3.7% vs SM22α^bright^ 21% ± 4%, N = 4, p = 0.395; retina SM22α^dim^ 40.7% ± 6.1% vs SM22α^bright^ 20.9% ± 4.8%, N = 3, p = 0.0635). That indicates that the SM22α^bright^ VSMCs are less (percentage) compared to SM22α^dim^ VSMCs (4-times in pial vessels, 1,3-times in parenchymal, 2-times in retina). The two VSMC subpopulations taken from different solid tissue origin are expressing the same percentage of ET_B_ receptor, not statistically significant (Fig 3C) (pial vessels SM22α^dim^/ET_B_^+^ 91.7% ± 2% vs SM22α^bright^/ET_B_^+^ 88.6% ± 3.3, N = 5, p = 0.45; parenchymal vessels SM22α^dim^/ET_B_^+^ 86% ± 3.7% vs SM22α^bright^/ET_B_^+^ 91.7% ± 3.3%, N = 4, p = 0.3; retina SM22α^dim^/ET_B_^+^ 92.2% ± 2.8 vs SM22α^bright^/ET_B_^+^ 88.8% ± 2.6%, N = 3, p = 0.42).

### Immunofluorescence microscopy

**Fig 4:**
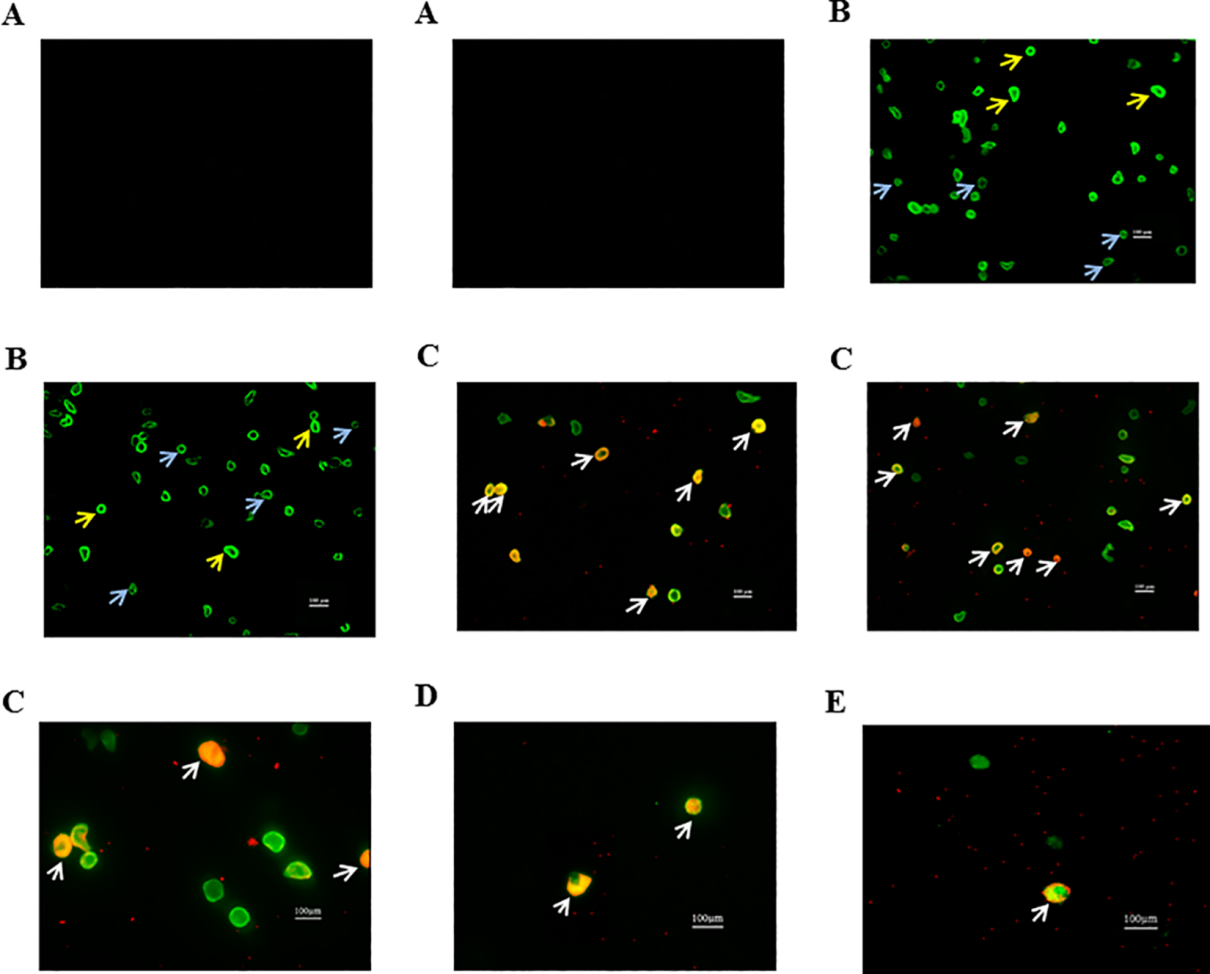
Representative pictures taken from fluorescence microscopy of isolated smooth muscle cells (positive for SM22α; green) and endothelin B receptor (positive ET_B_; red) from different tissue. (A) IgG SM22α and IgG ET_B_ (N = 3); (B) VSMC of pial vessels stained with SM22α, blue arrows-dim VSMC and yellow arrows-bright VSMC (N = 3); (C) VSMC of pial vessels stained with SM22α and ET_B_ (N = 3); (D) VSMC of parenchymal vessels stained with SM22α and ET_B_ (N = 3); (E) VSMC of retina vessels stained with SM22α and ET_B_ (N = 3). The white arrows indicate cells positive for both SM22α (green) and ET_B_ receptor (red).

Immunofluorescence microscopy confirmed the flow-cytometry results. We detected two subpopulations of VSMC and ET_B_ receptor localized on VSMCs. Fig 4A represents IgG for SM22α and IgG for ET_B_ receptor. Fig 4B shows two VSMC subpopulations (blue arrows indicate dim VSMC, SM22α^dim^, and yellow arrows indicate bright VSMC, SM22α^bright^) based on the expression of SM22α marker by smooth muscle cells taken from pial vessels. Fig 4C, 4D and 4E represent pictures of VSMCs expressing ET_B_ receptor taken from pial, parenchymal and retina vessels, respectively. The white arrows indicate the co-localization of SM22α (marker for VSMC) and ET_B_ receptor.

### Changes in subarachnoid hemorrhage stroke

**Fig 5:**
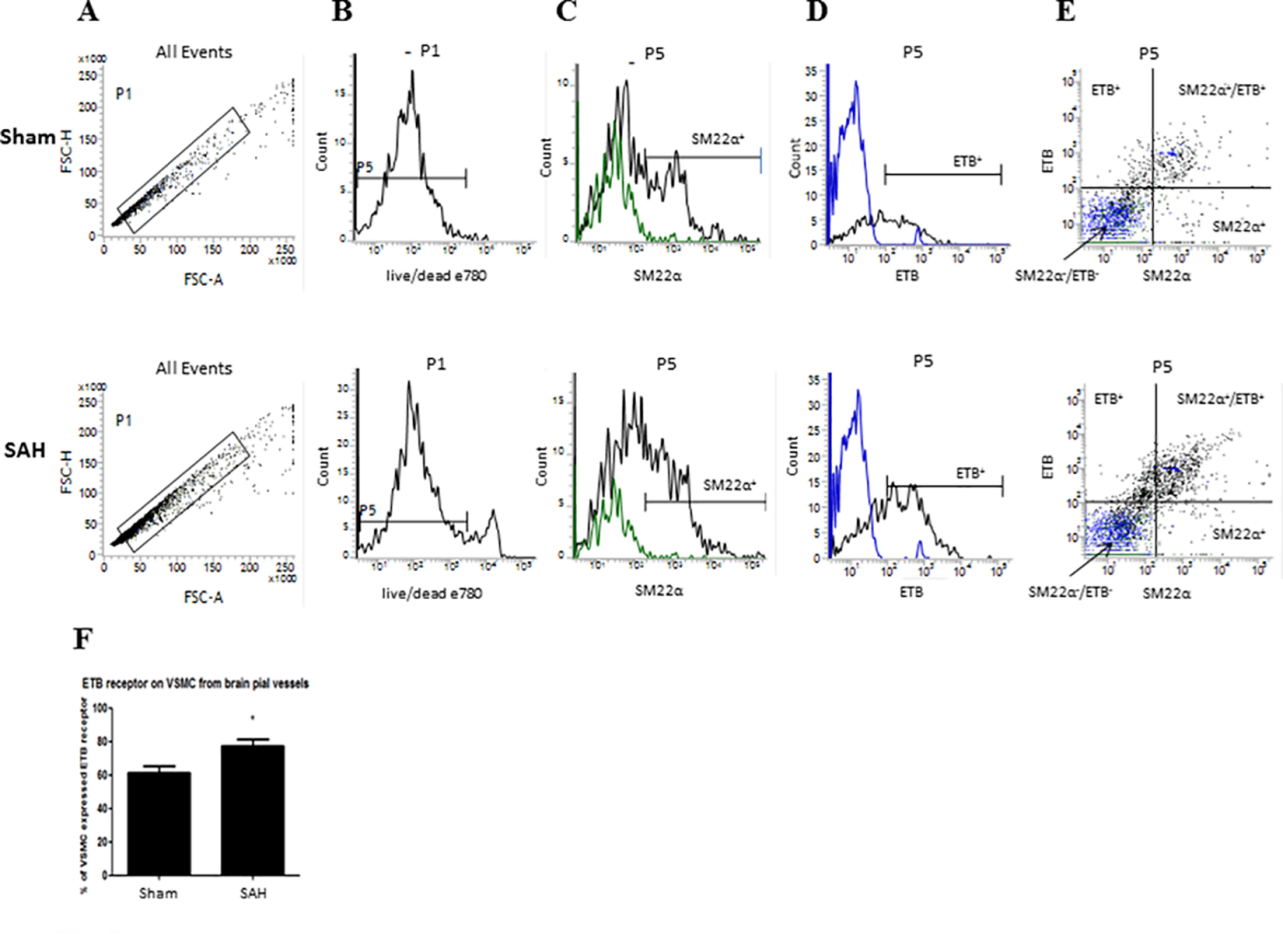
Changes in ET_B_ receptors expression on VSMC taken from pial vessels after induction of subarachnoid hemorrhage stroke. Representative traces taken from flow cytometry. (A) Dot plot histogram of single cells population according to FSC-H (H- high) vs. FSC-A (A- area scaling); (B) Representative histogram of negative single cells population for Fixable Viability Dye eFluor 780 (viable cells, live/dead); (C) Representative histograms demonstrating SM22α-positive events of viable cells suspension (log scale), the green line indicates the IgG for SM22α; (D) Representative histograms demonstrating ET_B_-positive events (log scale), the blue line indicates the IgG for ET_B_; (E) SM22α and ET_B_ events plotted on x, y axis, the green dots indicate the IgG for SM22α and the blue dots indicate the IgG for ET_B_; (F) summary data of the results (N = 6, *p<0.05).

The Fig 5 demonstrates the changes in expression of ET_B_ receptors on VSMC from pial vessels taken from control rats (sham) and rats induced by subarachnoid hemorrhage (SAH). In order to discriminate doublets and obtain single cells suspension, the FSC-H vs. FSC-A was correlated (Fig 5A). Fig 5B represents viable cells, which were further sub-gated and SM22α, ET_B_ were detected based on their fluorescence (Fig 5C and 5D), respectively. The expression of ET_B_ receptors on VSMC was measured. Furthermore, SM22α and ET_B_ events were plotted on x, y axis (Fig 5E). The top right quadrant shows double positive cells for both staining (SM22α^+^ and ET_B_^+^). The bar graph on Fig 5F demonstrates the summary data of changes in ET_B_ expression on VSMC after SAH. The results are statistically significant, sham 61.4% ± 4%, vs SAH 77.4% ± 4% (N = 6, p = 0.02), which confirm the immunohistochemistry results and supports previous work of our group [8].

## Discussion

In order to focus on translational research, it is crucial to develop in vitro approaches to elucidate mechanisms responsible for tissue and vessel pathology after brain injury and to localize the cellular expression versus whole tissue materials. Therefore, we decided to develop a single cell isolation procedure and a specific FACS based protocol for detecting pial, parenchymal and retinal VSMCs. We obtained high number of cell viability after the isolation process by Liberase TM Research Grade. Flow-cytometry technique is designed to use liquid tissue such as blood, cerebrospinal fluid, bone marrow. However, the flow cytometry can be used to study single cells isolated from solid tissue by a mechanical and enzymatic dissociation [6, 9–10].

We detected two subpopulations of VSMC by flow-cytometry. SM22α^dim^ population expressed less SM22α protein compared to SM22α^bright^ population. Moreover, there are more SM22α^dim^ cells (bigger population) in percentage compared to SM22α^bright^ cells population. We confirmed these flow cytometry results by immunofluorescence microscopy. The results of tissue taken from pial arteries between two subpopulations SM22α^dim^ and SM22α^bright^ were statistically significant, as they are pure arteries. VSMCs resided from parenchymal and retina vessels, which did not indicate to be statistical significant. However, there are additional VSMCs from capillary pericytes and venular VSMCs besides the arteriolar VSMCs. Thus the difference between these tissues could be due to the latter mixture which is supported by the work of Adhikari et al [11]. They distinguished two populations of VSMCs based on SM22α protein expression in adult aorta of male mice [11]. We did not see any statistical significant differences, in the expression of ET_B_ receptors between the two subpopulations of VSMCs (SM22α-positive) taken from different part of CNS. In order to validate our newly developed method we measured the expression of ET_B_ receptors on VSMC in sham and SAH rats. The results obtained are statistically significant and there is an increase of ET_B_ receptors expression in VSMCs of operated SAH versus sham animals. The findings were confirmed by immunohistochemistry of ET_B_ expression in vessel by previous work by our group [7, 8, 12]. Spray et al. demonstrated similar changes of ET_B_ receptors expression in another model of stroke (ischemic) [13]. The potential limitation of the flow cytometry method is the fact that the detection of dim fluorescence signals is limited by multiple factors such as the reagents, staining, and instrument parameters [14]. There is another method of VSMCs isolation for single cell suspension such as magnetic-activated cell sorting (MACS) [10]. The immunomagnetic cell separation procedures are applicable for our preparation of single cells suspension. MACS magnetic beads are conjugated to highly specific antibodies against a particular antigen on the cell surface. The separation of target cells can be achieved by positive selection, when the magnetically labeled cells are engaged within the column and unlabeled cells flow through. Next, the target cells are eluted from the column. Direct or indirect magnetic labeling can perform positive selection. Weber et al. demonstrated separation of fibroblasts, VSMCs and endothelial cells from the fetal rat ductus arteriosus by using the MACS separation process [15]. Interestingly, our method of single cell isolation can be used further for analysis in a microfluidic device. Qasaimeh et al. developed microfluidic system to capture multiple myeloma cells (a plasma cell cancer) based on anti-CD138 antibodies coated on the surface of 16 parallel microfluidic herringbone channels. Next, the cells of interest were labeled with fluorescently-tagged anti-CD138 antibodies and visualized by fluorescence or confocal microscopy. The microfluidic method has shown excellent correlation with flow-cytometry analysis. The fixed flow rate was 20 μl/min, the antibody concentration 20 μg/ml and cell number 2000 cells/ml. The microfluidic device is very sensitive as it is permitting detection of <10 cells/ml and the cell capture efficiency is 40–70% [16]. The benefit of implementation of our method with the microfluidic device is to directly visualize the isolated single cells. Moreover, this whole process will be more efficient in the amount of cells received because it will be not need to have any centrifugation steps during antibodies labeling. From a clinical point of view the use of microfluidics technique will minimize the required volume of tissue to be processed. However, the quantification of captured cells will need to be performed manually. In conclusion, we provide guidelines for isolation of VSMCs from adult rat brain pial, brain parenchymal and retina vessels. We have demonstrated that there are two subpopulations of VSMC (SM22α-positive), which express the same level of ET_B_ receptors on the cell surface. These findings suggest that for in vitro conducted experiments two subpopulations may be taken together or separately for analysis. However, we suggest further study of SM22α^dim^, SM22α^bright^ populations by sorting the cells and analyzing their genetic and functional properties. Finally, we acknowledge the practical usefulness of this method in studies of ischemic and subarachnoid hemorrhage stroke.

## Supporting information

S1 Fig Detection of two VSMC subpopulations and their ET_B_ receptors expression (parenchymal and pial vessels). **Representative traces taken from flow cytometry.** (A) Dot plot histogram for entire cell population after isolation. (B) Dot plot histogram of single cells population according to FSC-H (H- high) vs. FSC-A (A- area scaling); (C) Representative histogram of negative single cells population for Fixable Viability Dye eFluor 780 (viable cells, live/dead); (D) Representative histograms demonstrating SM22α-positive events of viable cells suspension (log scale), the green line indicates the IgG for SM22α; (E) Representative histograms demonstrating ET_B_-positive events of viable cells suspension (log scale), the blue line indicates the IgG for ET_B_; (F, G) Representative histograms demonstrating ET_B_-positive events of SM22α^dim^ and SM22α^bright^ cells (log scale) respectively, the blue line indicates the IgG for ET_B_.
(TIF)

S2 Fig Changes in ET_B_ receptors expression on VSMC taken from pial vessels after induction of subarachnoid hemorrhage stroke. **Representative traces taken from flow cytometry.** (A) Dot plot histogram for entire cell population after isolation. (B) Dot plot histogram of single cells population according to FSC-H (H- high) vs. FSC-A (A- area scaling); (C) Representative histogram of negative single cells population for Fixable Viability Dye eFluor 780 (viable cells, live/dead); (D) Representative histograms demonstrating SM22α-positive events of viable cells suspension (log scale), the green line indicates the IgG for SM22α; (E) Representative histograms demonstrating ET_B_-positive events of viable cells suspension (log scale), the blue line indicates the IgG for ET_B_; (F) Representative histograms demonstrating ET_B_-positive events of SM22α-positive cells (log scale), the blue line indicates the IgG for ET_B_.
SM22α-positive events were further sub-gated and ET_B_ receptor expression was measured.
(TIF)
