# Supplementary figures and images for: A novel multicolor flow-cytometry application for quantitative detection of receptors on vascular smooth muscle cells
Source: PLoS One. 2017 Oct 30;12(10):e0186504. doi: 10.1371/journal.pone.0186504 (PMC5662092; doi:10.1371/journal.pone.0186504)

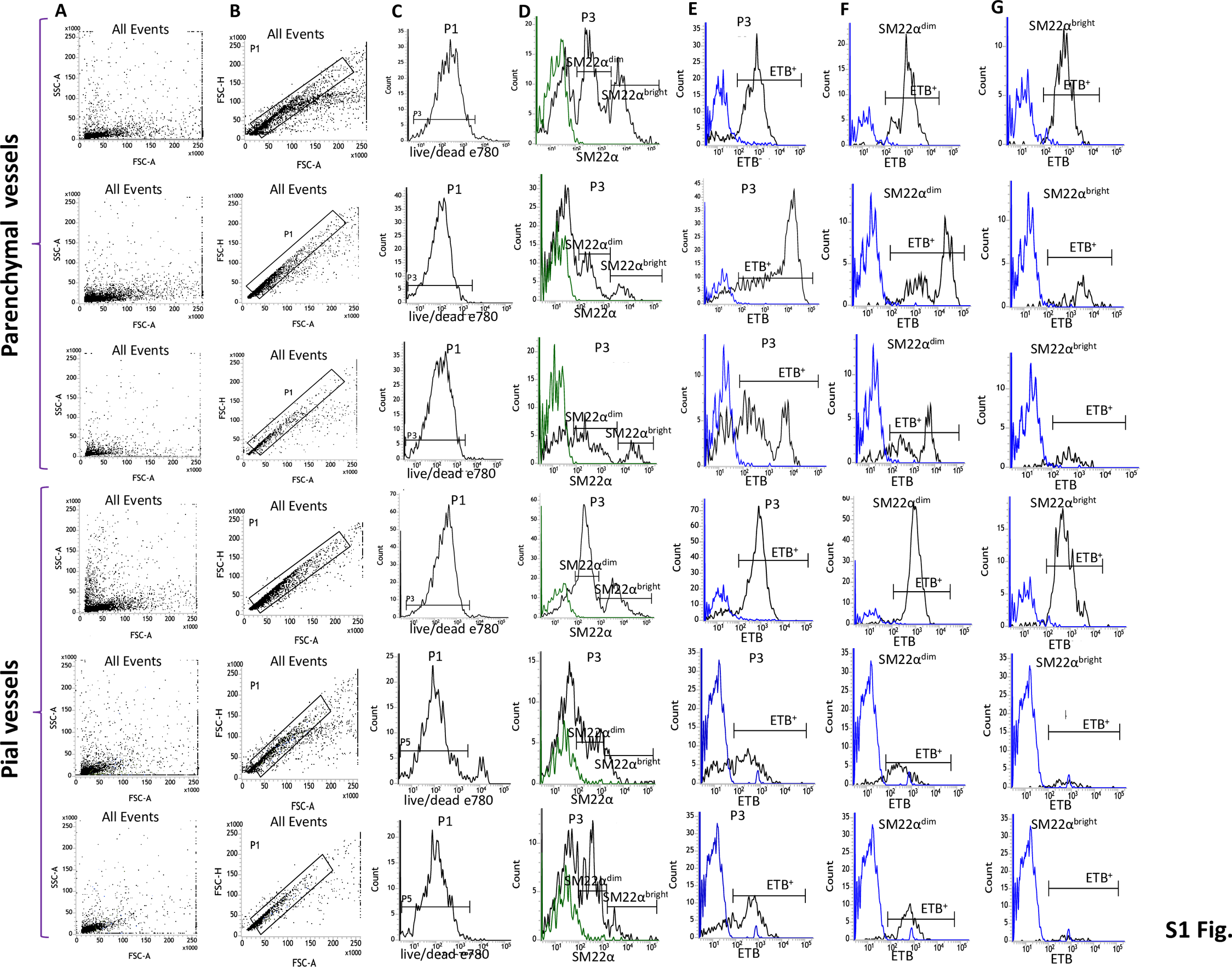

Supplement: S1 Fig — Representative traces taken from flow cytometry. (A) Dot plot histogram for entire cell population after isolation. (B) Dot plot histogram of single cells population according to FSC-H (H- high) vs. FSC-A (A- area scaling); (C) Representative histogram of negative single cells population for Fixable Viability Dye eFluor 780 (viable cells, live/dead); (D) Representative histograms demonstrating SM22α-positive events of viable cells suspension (log scale), the green line indicates the IgG for SM22α; (E) Representative histograms demonstrating ETB-positive events of viable cells suspension (log scale), the blue line indicates the IgG for ETB; (F, G) Representative histograms demonstrating ETB-positive events of SM22αdim and SM22αbright cells (log scale) respectively, the blue line indicates the IgG for ETB. (TIF) [file pone.0186504.s001.tif]

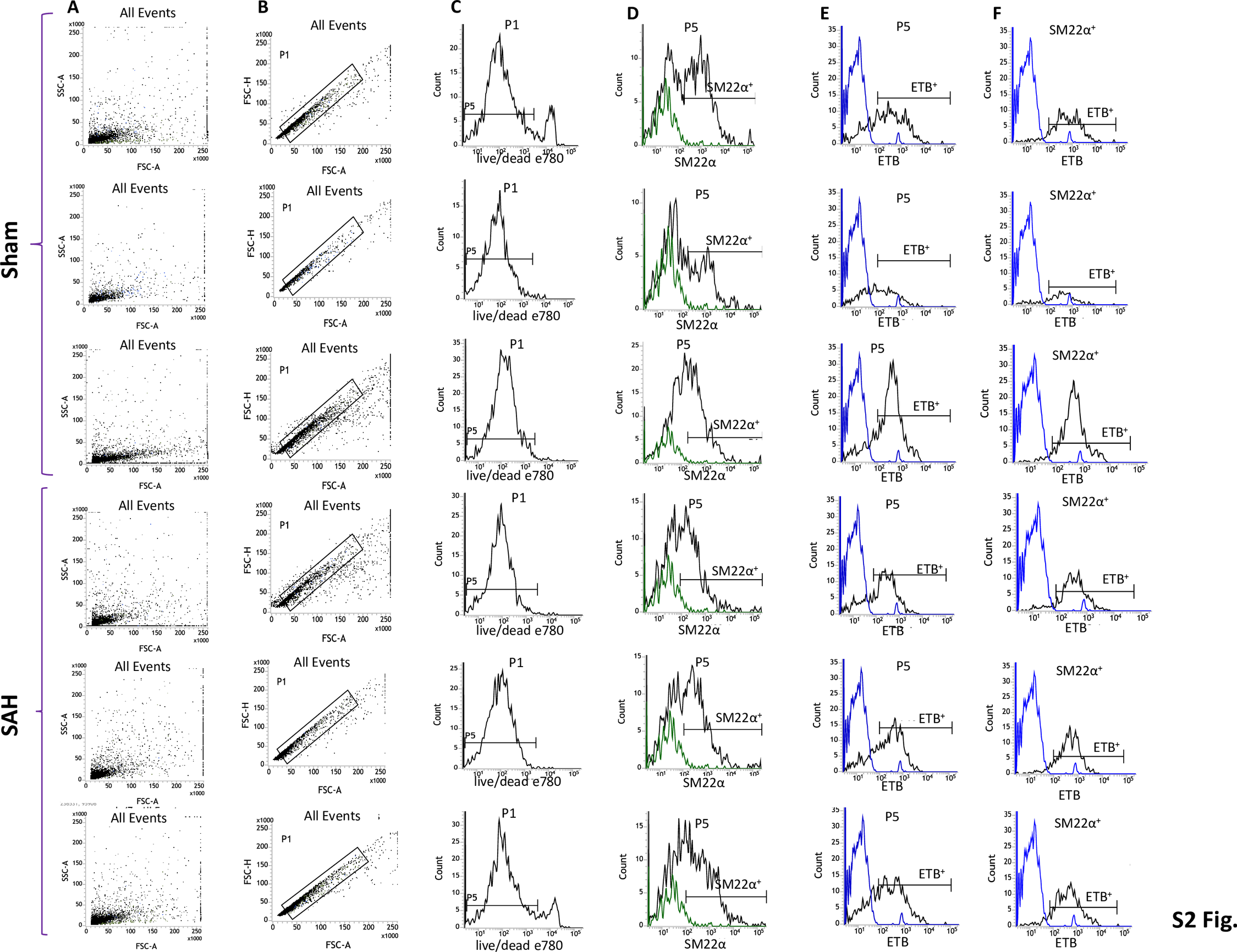

Supplement: S2 Fig — Representative traces taken from flow cytometry. (A) Dot plot histogram for entire cell population after isolation. (B) Dot plot histogram of single cells population according to FSC-H (H- high) vs. FSC-A (A- area scaling); (C) Representative histogram of negative single cells population for Fixable Viability Dye eFluor 780 (viable cells, live/dead); (D) Representative histograms demonstrating SM22α-positive events of viable cells suspension (log scale), the green line indicates the IgG for SM22α; (E) Representative histograms demonstrating ETB-positive events of viable cells suspension (log scale), the blue line indicates the IgG for ETB; (F) Representative histograms demonstrating ETB-positive events of SM22α-positive cells (log scale), the blue line indicates the IgG for ETB. SM22α-positive events were further sub-gated and ETB receptor expression was measured. (TIF) [file pone.0186504.s002.tif]
